# The complete chloroplast genome sequence of *Prunus glandulosa* Thunb. (Rosaceae)

**DOI:** 10.1080/23802359.2021.1982655

**Published:** 2021-09-30

**Authors:** Meihong Yi, Lixue Yang, Shiliang Zhou

**Affiliations:** a Key Laboratory of Sustainable Forest Ecosystem Management-Ministry of Education, School of Forestry, Northeast Forestry University, Harbin, PR China; b State Key Laboratory of Systematic and Evolutionary Botany, Institute of Botany, Chinese Academy of Sciences, Beijing, PR China; c College of Life Sciences, University of Chinese Academy of Sciences, Beijing, PR China

**Keywords:** *Prunus glandulosa*, Rosaceae, chloroplast genome, phylogeny

## Abstract

*Prunus glandulosa* Thunb. is an ornamental species in the genus *Prunus* (Rosaceae) mainly distributed in eastern China. It is often cultivated in gardens, roadsides, or shrub clusters. It looks like a cherry but resembles to prunes as well. We obtained the complete chloroplast genome of *P. glandulosa* using next-generation sequencing technology. The chloroplast genome is 158.078 bp in length with typical tetrad structure. It includes two copies of inverted repeats (IRs, 26.385 bp), a large single copy (LSC, 86.269 bp) and a small single copy (SSC, 19.039 bp). The total GC content is 36.7%, including 85 protein-coding genes (PCGs), 36 transfer *RNA* genes (tRNA), and eight ribosomal *RNA* genes (rRNA). The maximum-likelihood phylogeny using the full length of chloroplast genomes indicates that *P. glandulosa* is closer to prunes than to cherries.

*Prunus glandulosa* Thunb. is an ornamental species in the genus *Prunus* (Rosaceae) mainly distributed in eastern China. It is extensively cultivated worldwide for its beauty of abundant flowers. Prunus glandulosa (1929) is usually a small dwarf very similar to *P. japonica* and this group of species was classified into dwarf cherries (*Cerasus* subg. *Microcerasus*). However, the morphology of dwarf cherries is also very similar to prunes (*Prunus* s. s.). The phylogeny based on chloroplast genome has not been reported. Therefore, markers showed that the dwarf cherries do not have a close relationship to true cherries, instead, it is independent to either cherries or prunes. To test the taxonomic statuses of dwarf cherries and pinpoint their systematic position in *Prunus*, we determined the complete chloroplast genome of *P. glandulosa* and constructed the phylogeny of the dwarf cherries and other major lineages in *Prunus* subg. *Prunus* using the complete chloroplast genome sequences.

Total genomic DNA was extracted from the silica gel dried and clean leaves of *P. glandulosa* sampled from Wuhan Botanical Garden (China; N30°31′21.62″, E114°25′34.99″). Meanwhile, a sample (BOP002698) was corrected from the specimen housed in the herbarium of Institute of Botany, Chinese Academy of Sciences (PE). Total genomic DNA was extracted using modified cetyltrimethy lammonium bromide (mCTAB) method (Li et al. 2013). An Illumina DNA library was constructed using the total DNA and the DNA library was sequenced on Illumina HiSeq2500 platform for paired-end 150 bp reads (PE150). The genome is assembled using novoplasty (Dierckxsens et al. 2017, 2020) and annotated using PGA (Qu et al. 2019).

The newly assembled chloroplast genome (MW599984) of *P. glandulosa* is 158.078 bp in length with typical tetrad structure. It includes two copies of inverted repeats (IRs, 26.385 bp), a large single copy (LSC, 86.269 bp) and a small single copy (SSC, 19.039 bp). The GC contents of LSC, SSC, and IR regions are 34.5%, 30.4%, and 42.5%, respectively, and the whole genome is 36.7%. A total of 129 genes were annotated, including 85 protein-coding genes (PCGs), 36 transfer *RNA* genes (tRNA), and eight ribosomal *RNA* genes (rRNA).

To determine the taxonomic statuses of dwarf cherries and their systematic position in *Prunus*, we downloaded all chloroplast genomes of species in *Prunus* subg. *Prunus* and of *Prunus hypoxantha* (*Prunus* subg. *Padus*) as an outgroups in GenBank, aligned them with MAFFT (Katoh and Standley 2013), and constructed a maximum likelihood (ML) phylogeny (Figure 1) using IQ-TREE (Nguyen et al. 2015) using the best-fitting model K3Pu + F + I determined by ModelFinder (Kalyaanamoorthy et al. 2017). The phylogeny shows that the dwarf cherries are more similar to prunes than to true cherries and the dwarf cherries are actually dwarf prunes.

**Figure 1.**
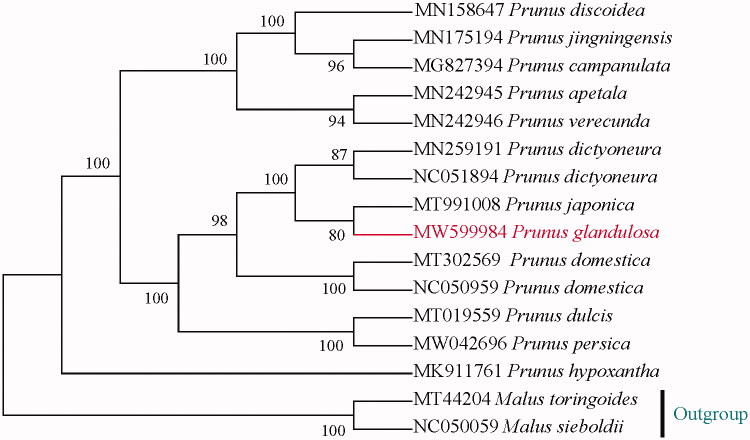
Phylogenetic tree based on plastid genomes using the ML method. Ultrafast bootstrap (UFBoot) values are shown above the nodes, with 1000 boot-strap replicates.

## Data Availability

The genome sequence data that support the findings of this study are openly available in GenBank of NCBI at (https://www.ncbi.nlm.nih.gov/) with the accession number is MW599984. The associated BioProject, SRA, and Bio-Sample numbers are PRJNA742221, SRR14982105, and SAMN19945336, respectively
